# Data Base Design with GIS in Ecosystem Based Multiple Use Forest Management in Artvin, Turkey: A Case Study in Balcı Forest Management Planning Unit

**DOI:** 10.3390/s90301644

**Published:** 2009-03-10

**Authors:** Hacı Ahmet Yolasığmaz, Sedat Keleş

**Affiliations:** 1 Artvin Çoruh University, Faculty of Forestry, Artvin, Turkey; E-mail: ahyol06@hotmail.com; 2 Karadeniz Technical University, Faculty of Forestry, Trabzon, Turkey; E-mail: sedatkeles06@hotmail.com

**Keywords:** ecosystem based multiple use forest management planning, geographic information systems, database, and land cover change

## Abstract

In Turkey, the understanding of planning focused on timber production has given its place on Multiple Use Management (MUM). Because the whole infrastructure of forestry with inventory system leading the way depends on timber production, some cases of bottle neck are expected during the transition period. Database design, probably the most important stage during the transition to MUM, together with the digital basic maps making up the basis of this infrastructure constitute the main point of this article. Firstly, the forest management philosophy of Turkey in the past was shortly touched upon in the article. Ecosystem Based Multiple Use Forest Management (EBMUFM) approaches was briefly introduced. The second stage of the process of EBMUFM, database design was described by examining the classical planning infrastructure and the coverage to be produced and consumed were suggested in the form of lists. At the application stage, two different geographical databases were established with GIS in Balcı Planning Unit of the years 1984 and 2006. Following that the related basic maps are produced. Timely diversity of the planning unit of 20 years is put forward comparatively with regard to the stand parameters such as tree types, age class, development stage, canopy closure, mixture, volume and increment.

## 1. Introduction

Forest resources in the world were planned and operated in the axis of timber production until the early 1960s. Population growth and appearance of the metropolises as well as the developments in industry and information systems have given rise to the increase and diversity of human needs. So that the ways of utilizing forests have changed and they have taken the first place in the scope of economic and social values. Multiple forest use philosophy being carried out the early 1990s has reached a level threatening forest sources by backing up the legal and technical infrastructures. In the period of last 20 years, anxieties about the decrease in biological diversity, climate changes, drought, nature and especially forest ecosystems as well as information flow, increase in the effectiveness of civil movements of nature protection have centered the ecosystem planning in the world agenda. Each country adapts the philosophy of forest sources management to forestry in its own entity even under varied names, by supported from international conventions and processes [1–8].

During the stated period, the world countries took the first pace in Rio, 1992 with the convention of Biological Diversity (BD) in search of a common solution. Moreover, they accelerated the solution via processes in the continental scale. Turkey signed BD Convention in 1996 and it supports the solution search with its forests spreading around two continents by including the processes of Pan Europe and Near East because of its geographical position. Forests in the country were planned and operated according to the classical planning approach which focused on timber production from 1963 to 1995, a period of transition to planned forestry. Up to now, the search on planning the forests resources beyond the classical approach has appeared in all times in the name of model plans. In Turkey, the first samples of decisions and applications agreed in the international conventions and processes are Multiple Use Forest Managements belonging to the planning units of the Regional Directory of Istanbul in 1995. After this date, various planning systems have been suggested in different names (FRIS Project etc.); however the basic philosophy has the functional planning or in other words Multiple Use Management [9–13]. In Turkey, the sample forest management plans applied or still being carried out, are trials of forestry that are Based on timber production, that don’t question the infrastructure of traditional forestry and that have deficiencies in application and supervision stages.

During the period of last 5 years, Multiple Use Management approach has taken the name of Ecosystem Based Multiple Use Forest Management (EBMUFM) by modifying and developing together with the biological diversity supports. Besides, it has been approved by the most of foresters, managers and scientists in Turkey. In the near future, Forest Management Plans will be arranged with this approach. The formation process of the Ecosystem Based Multiple Use Forest Management made up of basically seven stages [14, 15].

The formation process of the Ecosystem Based Multiple Use Forest Management to be established on a infrastructure with a focus on timber production shall cause a great many problems at the application stage. In this article, especially spatial database formation, the second stage of Ecosystem Based Multiple Use Forest Management, is emphasized. In this context, data structures of the sample forest management plans with domestic and foreign financial supports and those prepared in more than 40 years have been examined. Moreover, their facilities, and the blocked points have been questioned as well as how the data infrastructure of the EBMUFM should be has been considered. Geographical databases have been established by storing the data of forest management plans relating the past and present (1984 and 2006), belonged to Regional Directory of Artvin Forest, Borçka Forest Enterprise, Balcı Planning Unit. In addition to that, related supports and a data dictionary were prepared. The Forest Management plan in 1984 was prepared in the axis of timber production whereas the one in 2006 was prepared in the shade of multiple use management approach. However, it is clearly seen that even in the new plan, the approach with a focus on timber production is dominant. Numerical geographic database in 1984 was produced in the scope of this study. The basic numerical database (stand map) was provided from the Principal of Artvin Planning and Project Branch and the geographical database were developed by being examined once more. Development and modification of Balcı planning Unit during a period of 20 years have been evaluated comparatively with regard to the stand parameters such as mixture of tree types, canopy closure, age, growing stock and increment, and development stage.

## 2. Materials and Methods

### 2.1. The Study Area: Balcı Forest Planning Unit

**Figure 1.**
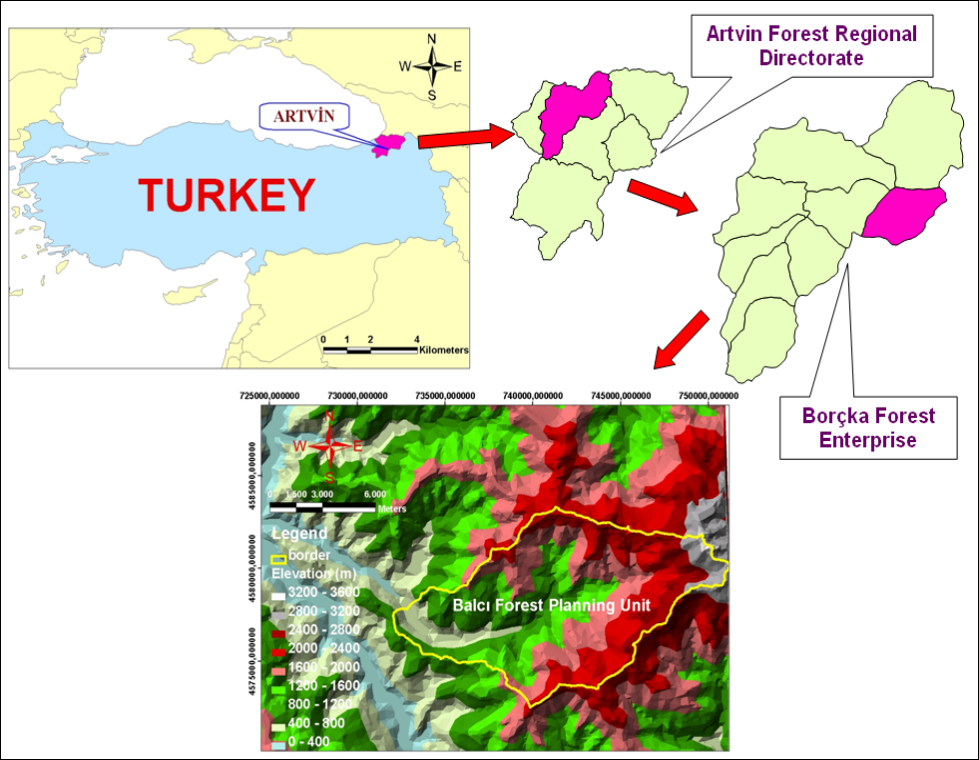
Location of the study area: Balcı Planning Unit in Regional Directory of Artvin Forest in Turkey, and Digital Elevation Model.

The study area is the Balcı Forest Planning Unit (BFPU) located in the northeast of Turkey characterized by a dominantly steep and rough terrain with an average slope of 58% and altitude from 340 to 3414 m above sea level. It extends along UTM ED 50 datum 37. zone 732000–751000E and 4572000–4583000N on the northeastern Black Sea region of Turkey (Figure 1). The total area is 10,806.13 ha. The vegetation type is forest vegetation and the dominant tree species of the vegetation are Picea orientalis (L.) Link, Fagus orientalis Lipsky, Abies nordmanniana (Stev.) Spach subsp. nordmanniana, Castanea sativa, Tilia rubra subsp. caucasiaca, Alnus glutinosa subsp. barbata, Pinus sylvestris L., Carpinus betulus L. Although no comprehensive study about plant sociology in the study area has been done, seven various tree species such as Quercus pontica, Quercus petraea and Ostrya carpinifolia Scop as well as 14 different shrubs and brushes and 18 plant types taking into the group sporadically that is not standing on their own have been detected and recorded. Mean annual temperature of the study area is 13.5°C, and mean annual precipitation is 1009.3 mm. Main soil types are sandy clay loam, clay loam and sandy loam [16]. In the planning unit, Balcı Village and its neighborhoods take place. While its population was 705 in 1990, this number decreased 532 in 2000 and 450 in 2007 [17–19]. Public’s means of living are generally provided from forest works (production, transportation, road construction and forestation). Besides, hazelnut, tea and corn agricultures as well as livestock production necessary enough to meet the basic needs are carried out. Beekeeping is very common and well-known around the district, moreover the hives peculiar to the region are settled in tall trees or in the rock hollows. Because of the high pitched topography and harsh winter conditions, in Balcı district the migration from villages to the city have been going on around Artvin where industrial investments are limited and living conditions are hard [16, 20].

### 2.2. Database Design Based On the Ecosystem Based Multiple Use Forest Management

**Figure 2.**
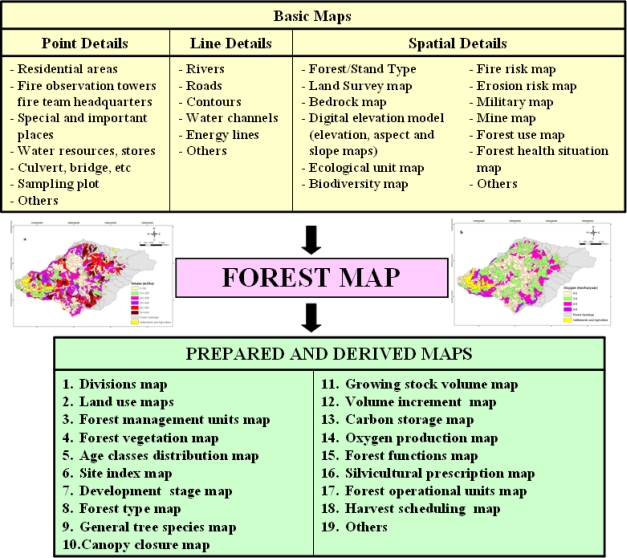
EBMUFM data base design model in Turkey.

The purpose of the EBMUFM is to produce more than one value and service in the same forest area and to offer it to public service with no damage to the health and integrity of forest ecosystem. For this reason, first of all a forest function map indicating economic, ecologic and numeric values as well as making up the basic skeleton of the system is designed. During the preparation of forest functional map, more than 20 basic numeric values made up of details such as point (sampling plots, maps of residential plots etc.), line (rivers, roads, energy lines, etc.) and polygon (stand types map, compartments map, etc) are used. By utilizing the forest functional map, operational aims, protection purposes and silvicultural prescription units are determined and silvicultural prescriptions are prepared. The forest model is established and than solved according to the plan strategies by using the techniques of operational research and alternative forest management strategies are developed. A much more comprehensive forest map is provided by entering the support related to each alternative willingly or fixed alternative (as a harvesting schedule). The fixed forest map can create 17 and much more various maps by using the table of main quality data. Each one of the produced maps includes data about planning unit for some of the indicators and criteria of sustainable forest management that is carried on internationally and nationally (Figure 2).

Together with this, data entering standard needs to be formed in order to prevent data loses in combining the planning units to the immediate planning units of forest maps, in coinciding with various basic maps and in producing new maps. At this point, the basic principles to be considered are data types being comprehensible by the users, their occupying minimum place in data storage and not facing data lose in transforming an exporting to various software.

In the establishment of database related to the study area, Geographical databases have been established by storing the data of forest management plans relating the past and present (1984 and 2006), belonged to Balcı Planning Unit. In addition to that, related coverage and a data dictionary were prepared. The GIS presentation in Balcı was accomplished using the following GIS data; forest cover type maps for Balcı. Source scale of these maps was 1:250,000, based on the National Map Accuracy Standard for 1:250,000 maps. Forest cover type maps of case study areas (Balcı) in 1984 were firstly digitized and processed using Arc/Info version 9.2 GIS with a maximum root mean square (RMS) error under 10 m and spatial database established. The basic database (only stand type map) in 2006 was provided from the Regional Directory of Artvin Forest-Planning and Project Coordinator and the geographical database were developed by being examined once more. Spatial databases consists of stand type, dominant tree species, mixture, canopy closure, forest development stages, age class, basal area and stand type area. The stand type volume and increment was added to the database. Development and modification of Balcı planning Unit during a period of 20 years have been evaluated comparatively with regard to the stand parameters such as mixture of tree types, canopy closure, development stage, stand age and growing stock.

## 3. Results and Discussions

When the study area is evaluated in terms of forest areas, a decrease in productive forest areas (in 1984; 5571.96 hectares, in 2006; 5343.51 hectares) is seen (−228 hectares) while an increase in degraded forest areas (+ 38 hectares) is observed. Based on the development in techniques of forestry inventory, 35 various stands were described in 1984 while this number was 83 in 2006. The smallest sub compartment number, subjected to planning and silvcultural units, was 795 in 1984 while it was 1194 in 2006. There has been no change in the limits and numbers of compartments that are control units (Table 1).

**Figure 3.**
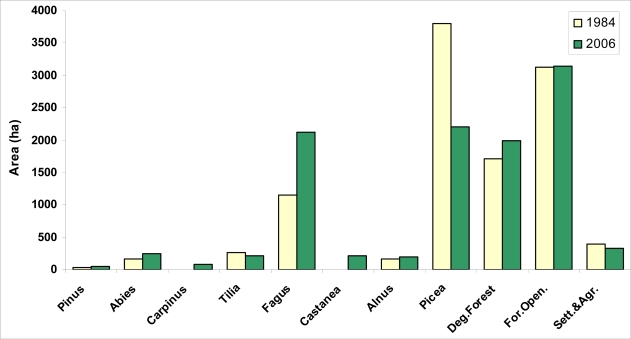
Area distribution of the dominant tree species in 1984 and 2006 in the planning unit.

**Figure 4.**
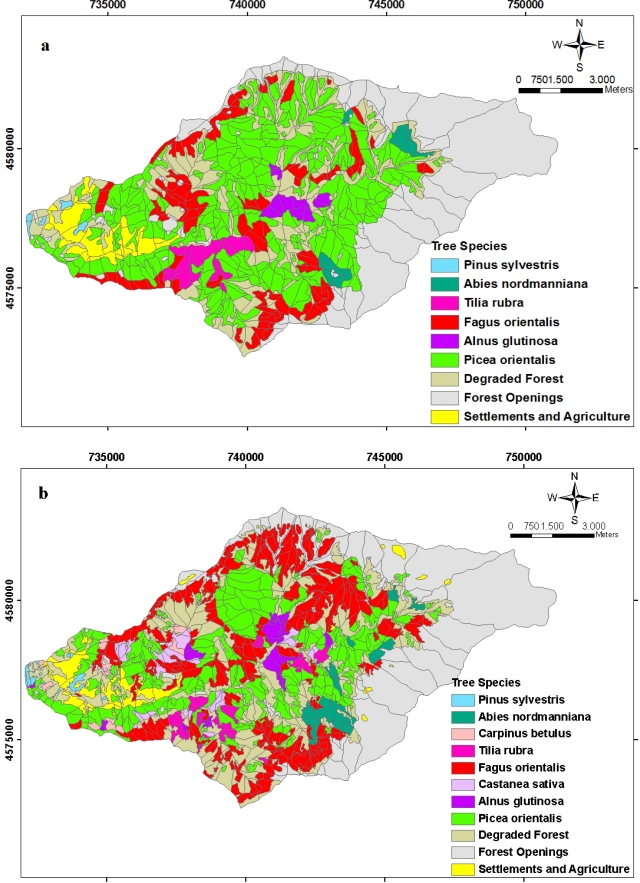
The spatial distribution of the planning unit related to the dominant tree species **(a)** in 1984 and **(b)** in 2006.

Figure 3 shows the area distribution of dominant tree species in 1984 and 2006. In Figure 4, the spatial distribution of tree species in 1984 and 2006 is seen. Among the dominant tree species (a tree species with the highest volume rate among the mixed stands and the one with more than 10 % regarding the volume), Picea orientalis is the one with the most area. While Picea orientalis was 3803.96 (68.27%) hectares in 1984; it covered an area of 2206.31 (41.29%) hectares according to the data of 2006. The second tree species with the most area on the other hand is Fagus orientalis. While it was 11449. 47 (20.63%) hectares in 1984, it showed the distribution of 2131 (39.89%) hectares in 2006. When the areas of both tree species in 1984 and 2006 are compared, total area of Picea orientalis decreases as total area of Fagus increases. Another striking issue at this point is that during 22 years Carpinus betulus tree type has come into being with 87.7 hectares (Figure 4).

**Figure 5.**
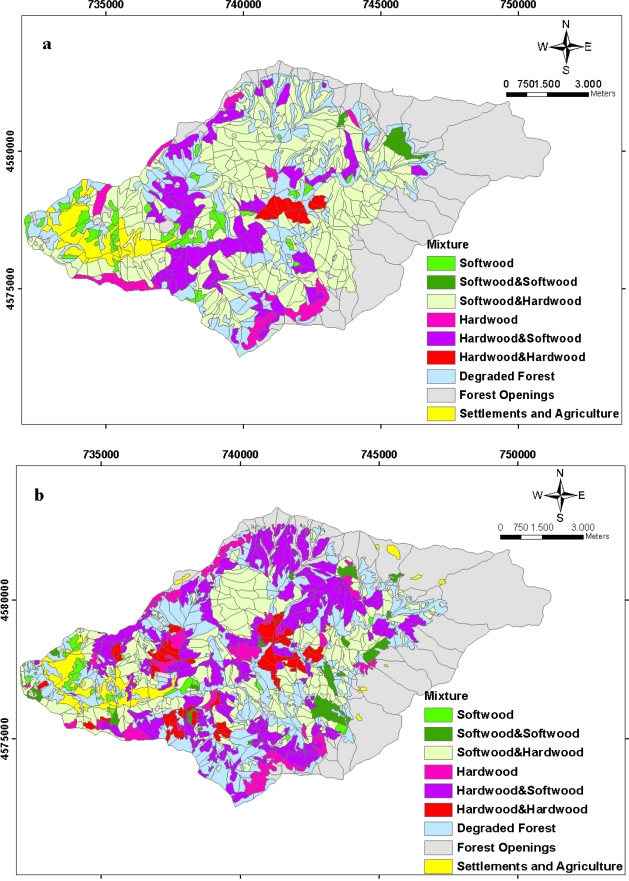
The spatial distribution of the planning unit related to the forest types **(a)** in 1984 and **(b)** in 2006.

**Figure 6.**
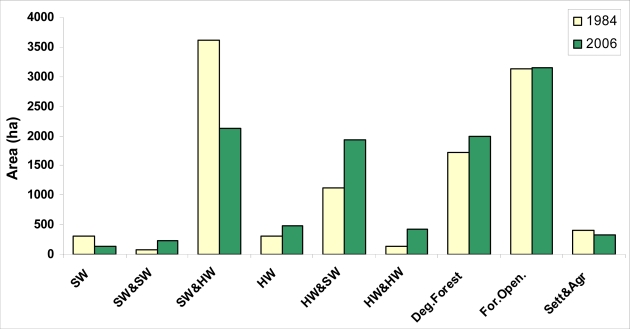
Area distribution of the forest types in 1984 and in 2006 in the planning unit.

Spatial distribution of the forest types between 1984 and 2006 with regard to the study area is seen in Figure 5. Area distribution of the forest types during the period of 22 years is given in Figure 6. The study area is mostly made up of mixed stands of the hardwoods and softwoods. Most of the study area is made up of mixed stands including the coniferous. It has been found out that as is dominant tree species, there is an increase in hardwood stands (pure and mixed) (in 1984; 28.2%; in 2006 46.25%), whereas there is a decrease in softwood stands (pure and mixed) (in 1984 71.80%; in 2006 53.75%) (Figure 6).

**Figure 7.**
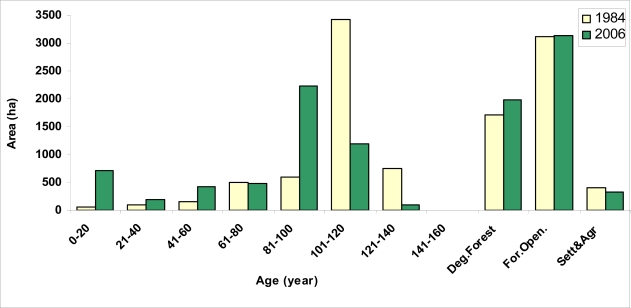
Area distribution of age classes belonged to the forest ecosystem in the planning unit in 1984 and 2006.

**Figure 8.**
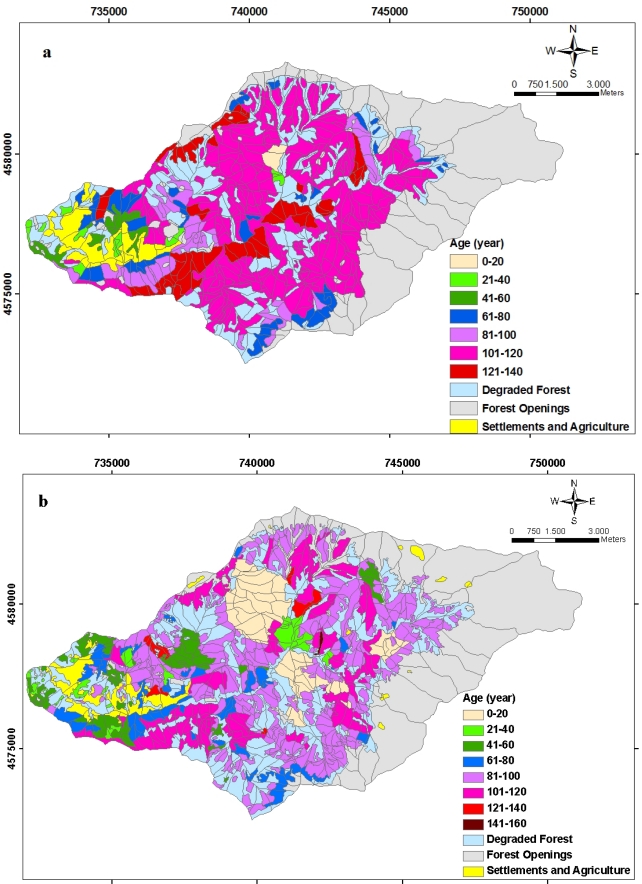
The spatial distribution of the planning unit related to the age class distribution **(a)** in 1984 and **(b)** in 2006.

The age class area distribution of planning unit between 1984 and 2006 is seen in Figure 7. According to Figure 7, as to the forest management data, 33.63 % of the productive forest areas are under the age of 80, 42.3 % are between 80 and 100, and 24.07 % are above the age of 100. Therefore, it can be stated that old growth forest potential of Balcı forests, which indicate seed rooted and natural, is high (Figure 8).

**Figure 9.**
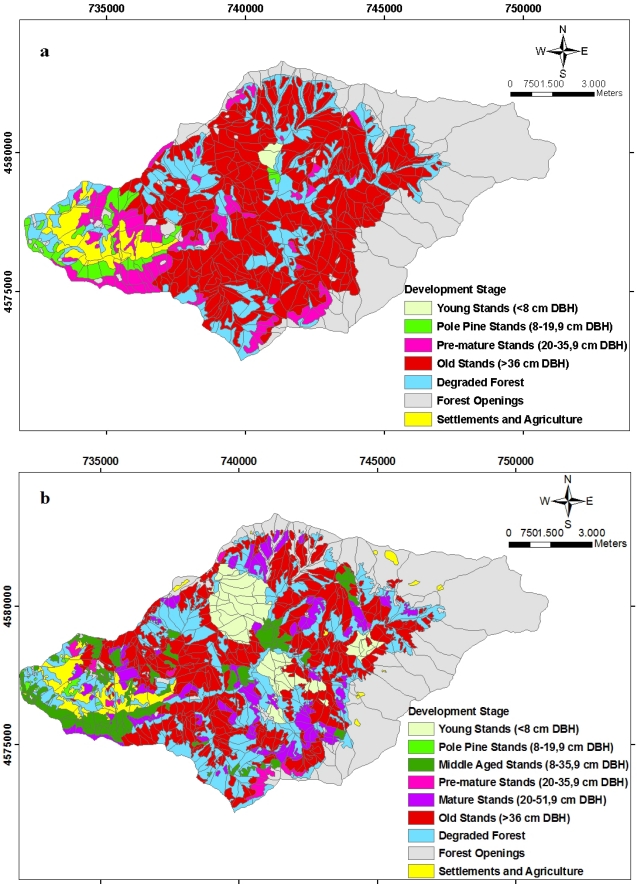
The spatial distribution of the planning unit related to the development stage **(a)** in 1984 and **(b)** in 2006.

In Figure 9, spatial distributions of the development stages of forest ecosystems in 1984 and 2006 are shown. As is seen in the figures, during the 22-year study period of development stages, some inconsistencies are seen in transition from one period to another and the stands made up of trees with thick diameters (> 52 cm) cover considerable area (55.33 %).

**Figure 10.**
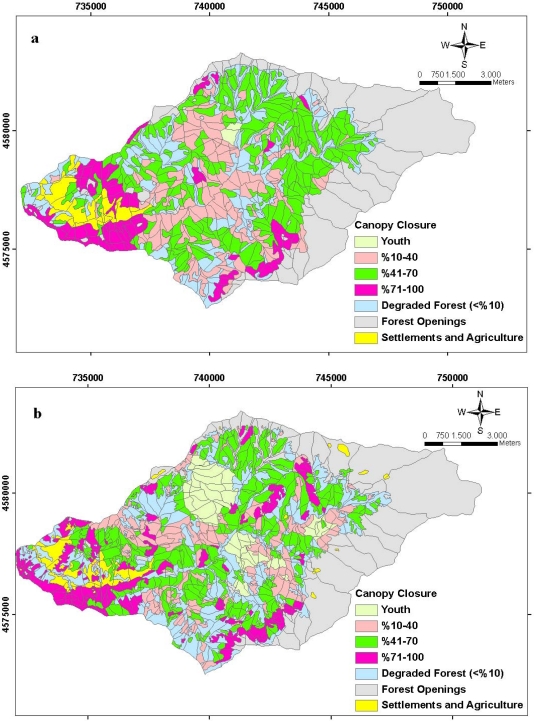
The spatial distribution of the planning unit related to the forest canopy closure **(a)** in 1984 and **(b)** in 2006.

The spatial distribution of forest canopy closure related to the forest ecosystem of study area is seen in Figure 10. When the canopy closures during the period of 22 years are evaluated, the area amount of young stands was approximately 50 hectares while that number reached about 714 hectares in 2006. While the areas with canopy closures at 10–40 % and 41–70 % decrease in transition from one period to another, the amounts of canopy closure areas at 70–100 % rose from 933 hectares in 1984 to 1085 hectares in 2006.

**Figure 11.**
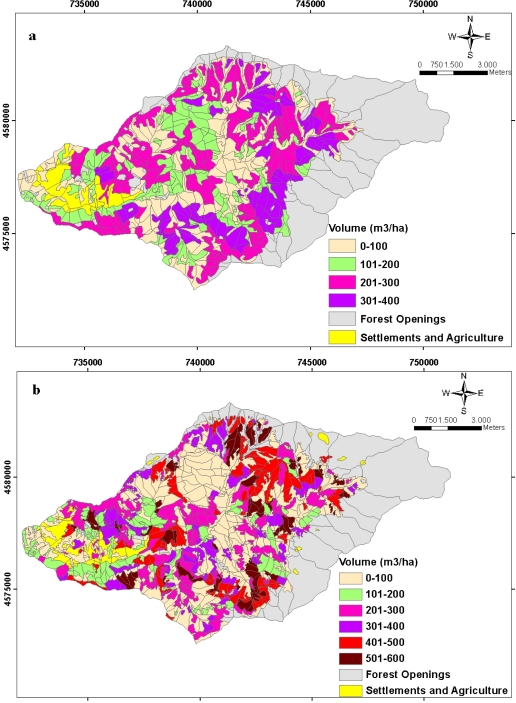
The spatial distribution of the planning unit related to the amounts of volume **(a)** in 1984 and **(b)** in 2006 in unit area.

**Figure 12.**
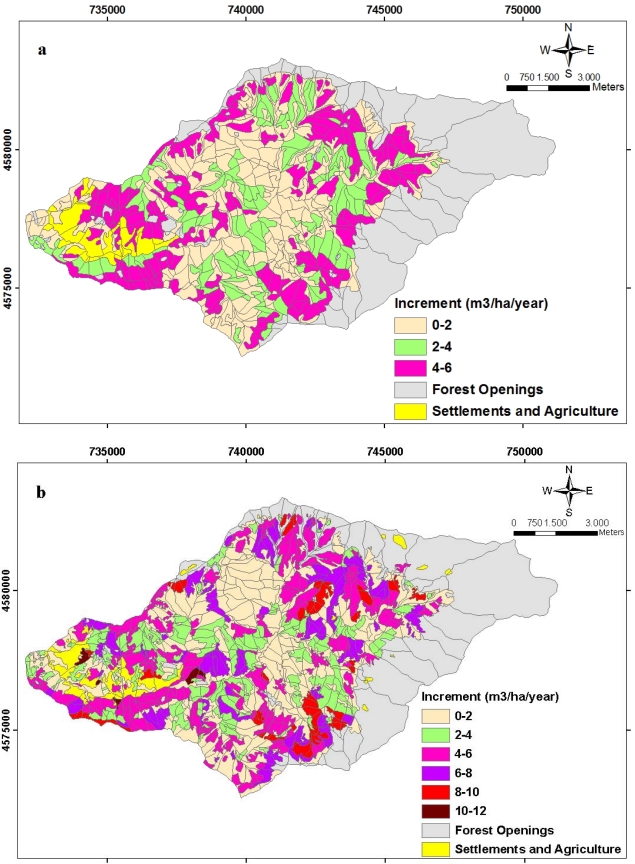
The spatial distribution of the planning unit related to the amounts of increment **(a)** in 1984 and **(b)** in 2006 in unit area.

The spatial distributions of growing stocks and the increments in a unit hectare belonged to Balcı Planning Unit in 1984 and 2006 are indicated respectively in Figure 11 and Figure 12. Total annual increment of forest ecosystem in the study area was 20615.638 m3/per year while this number was estimated as 24408.002 m3/per year in 2006. Total growing stock values were estimated in 1984 as 1,344,455.828 m3 whereas this number was 1,532,288.698 m3 in 2006. While Picea orientalis constituted a great part of both the increment and the growing stock in 1984, in 2006 this changed into as Fagus orientalis.

The basic reason of the changes and decrease in softwood areas as well as the inconsistency in timely transition regarding other stand parameters between 1984 and 2006 is damage of insects [21]. When the former forestry applications were examined, those were found out that extraordinary proceeds allowable cuts were taken (93 % of annual allowable cuts-10525 m3/year) in the direction of softwoods (especially Picea orientalis) and that the forest structure changed depending on the damage caused by bark beetles (Dendroctonus micans, Ips typographus and Ips sexdentatus). Unsuitable topography, not being able to take enough sampling plots and individual rough measuring mistakes are also other effective factors. During the renewal of plan, there was no possibility to compare the past and the new ones because there were no database and digital maps of 1984, as a result likely mistakes in the present situation couldn’t be examined before the plan was renewed.

In Turkey, especially in the Black sea Region, the fact that cadastre and ownership problems not being able to be solved is the basic reason of the changes in the areas without forest. There exist 7.8 million forest villagers living within and around the forests by making use of the forest sources in 20974 forest villages (In 1985 this was 10.2 million). Economic conditions of the people living in these areas are rather low, that is they make their living generally from forestry, partially from agriculture and livestock. Forest villagers constitute a domination factor on the forest areas in both positive and negative ways. Their positive effects are their socio-cultural life styles in or around the forests of their residential areas, the lack of compromise and forming a resistance against the technical forest application towards the forest because of utilizing the ecological and economic values and services (such as festival, bee stands, flood, rolling of rocks). Their negative effects on the other hand are, opening the fields for agriculture, illegal cutting and burning. The district is a place losing its population because of the physical deficiencies. For that reason, recently the social pressure effect in negative way has decreased. As is seen in the tables, figures and graphics; parallel to the decrease in population, there has become a decrease of approximately 65 hectares in residential and agricultural areas.

## 4. Summary and Conclusion

In Turkey, during the preparation of forest management plans, the comparison of the past and future was made partially by examining graphics and tables after the prior calculations in the past because there were no geographical databases. Initiating from sub compartment and compartment with the smallest database design, the evaluation in each scale will be easier in such issues as planning unit, forest enterprises, regional directories and forests in the whole Turkey. Moreover, database design is crucially important for various companies and facilities, private enterprises and non-governmental organizations (NGOs) in order to evaluate the produced data through their own purposes consistent to the software they use. Turkey needs to develop a comprehensive forest database related to the country facts while it is still at the beginning of the way. As there are no or limited numerical values about the ecological and social benefits offered by forests, together with the biological diversity, leading the way, they can not be transferred to geographical databases. The evaluations in that sense can be fulfilled indirectly by initiating from stand parameters. However, all numerical data obtained from flexible databases can be updated by being added afterwards.

It is anticipated that the forest cadastral, one of the basic problems of Turkey, will be solved in the next five years. The basic reason of the changes in residential and agricultural areas is that the ownership in the forest, residential and agricultural areas hasn’t become definite yet. Especially 7.8 million forest villagers living in or around the forest make their living from the forest. Recently, the traditional life styles of Turkish people, especially of the young population, have shown a transition from rural areas/life to urban areas/life parallel to the changes in technological and informational fields. Therefore, the populations of the villages have reduced; average age has considerably increased in the remaining ones. Parallel to the decrease in young population in the villages, a recession in agriculture and livestock sectors has been seen. Especially, as the areas that are within or around forests and used for agriculture and livestock before, are left on their own; they have become forest areas via the seeds coming from the edges. During the last five years in which cadastre has gained importance, as many such areas have lost their characteristics of being pasture and agricultural sites, they are recorded on forest maps as forest areas during cadastre procedures.

In order to anticipate the future of forests in the country, new plan data which are prepared by multiple use management, as well as the plan data of about 40 years need to be recorded in geographical databases. In that way, models of spatial forest ecosystem indicating the dynamics of forest ecosystem which changes according to silvicultural prescriptions and natural events shall be produced. Short-term and long-term research projects about the difficult positions described in this context need to be developed in order to achieve Ecosystem Based Multiple Use Forest Management successfully. Universities as in the leading position, researchers, managers, administrators, NGOs, foresters and all the related groups are considerably responsible for this issue.

## Figures and Tables

**Table 1.** Comparison of the situations in the past and present with regard to the general features of the study area in BFPU.

**General Features**		**1984**	**2006**

Forest Management Planning Units		Three (timber production-beech and spruce-protection)	Two (timber production, hydrologic)

The number of stand type (unit)		35	83
Compartment (unit)		252	252
Stand number (unit)		254	420
Sub compartment number (unit)		795	1194
The smallest patch (hectare)		0.556	0.305
The biggest patch (hectare)		752.704	737.379

Forest area (ha)	Productive	5571.96	5343.51
	Degraded Forest	1717.59	1983.55
	Total	7289.55	7327.06

Non-forest area (ha)	Forest Openings	3121.39	3144.23
	Sett.&Agr.	400.92	334.85
	Total	3522.31	3479.08

Total (ha)		10811.86	10806.14
